# Factors associated with cancer treatment resumption after ICU stay in patients with solid tumors

**DOI:** 10.1186/s13613-024-01366-3

**Published:** 2024-08-31

**Authors:** Soraya Benguerfi, Ondine Messéant, Benoit Painvin, Christophe Camus, Adel Maamar, Arnaud Gacouin, Charles Ricordel, Jean Reignier, Emmanuel Canet, Julien Edeline, Jean-Marc Tadié

**Affiliations:** 1 CHU Rennes, Service de Maladies Infectieuses et Réanimation Médicale, Hôpital Pontchaillou, Université de Rennes 1, 2, rue Henri Le Guilloux, Rennes cedex 9, 35033 France; 2 Laboratory “Movement, Interactions, Performance” (EA 4334), Faculty of Sport Sciences, University of Nantes, 25 Bis Boulevard Guy Mollet, BP 72206, Nantes Cedex 3, 44322 France; 3 Service d’Hématologie Clinique, Hôpital Pontchaillou, CHU Rennes, Université de Rennes 1, 2, rue Henri Le Guilloux, Rennes cedex 9, 35033 France; 4 CHU Rennes, Service de Pneumologie, 2 Rue Henri Le Guilloux, Rennes, 35033 France; 5 INSERM, OSS (Oncogenesis Stress Signaling), UMR_S 1242, CLCC Eugene Marquis, Univ Rennes 1, Rennes, 35000 France; 6 CHU Nantes, Service de Médecine Intensive Réanimation, Nantes Université, 1 Place Alexis Ricordeau, Nantes Cedex 01, 44093 France; 7 CLCC Eugène Marquis, Service d’Oncologie Médicale, Université de Rennes 1, COSS (Chemistry Oncogenesis Stress Signaling), UMR_S 1242, Rennes, France; 8 INSERM, Microenvironment, Cell Differentiation, Immunology, and Cancer-UMR_S1236, Établissement française du sang Bretagne, Université de Rennes 2, Rennes, F-35000 France; 9 CIC 1414, Rennes, France

**Keywords:** Neoplasms, Intensive care unit, Cancer treatment, Outcome, Solid tumors

## Abstract

**Background:**

Post-intensive care syndrome could be responsible for inability to receive proper cancer treatment after ICU stay in patients with solid tumors (ST). Our purpose was to determine the factors associated with cancer treatment resumption and the impact of cancer treatment on the outcome of patients with ST after ICU stay.

**Methods:**

We conducted a retrospective study including all patients with ST admitted to the ICU between 2014 and 2019 in a French University-affiliated Hospital.

**Results:**

A total of 219 patients were included. Median SAPS II at ICU admission was 44.0 [IQR 32.8, 66.3]. Among the 136 patients who survived the ICU stay, 81 (59.6%) received cancer treatment after ICU discharge. There was an important increase in patients with poor performance status (PS) of 3 or 4 after ICU stay (16.2% at admission vs. 44.5% of patients who survived), with significant PS decline following the ICU stay (median difference − 1.5, 95% confidence interval [-1.5-1.0], *p* < 0.001). The difference between the PS after and before ICU stay (delta PS) was independently associated with inability to receive cancer treatment (Odds ratio OR 0.34, 95%CI 0.18–0.56, p value < 0.001) and with 1-year mortality in patients who survived at ICU discharge (Hazard ratio HR 1.76, 95%CI 1.34–2.31, p value < 0.001). PS before ICU stay (OR 3.73, 95%IC 2.01–7.82, p value < 0.001) and length of stay (OR 1.23, 95%CI 1.06–1.49, p value 0.018) were independently associated with poor PS after ICU stay. Survival rates at ICU discharge, at 1 and 3 years were 62.3% (*n* = 136), 27.3% (*n* = 59) and 17.1% (*n* = 37), respectively. The median survival for patients who resumed cancer treatment after ICU stay was 771 days (95%CI 376–1058), compared to 29 days (95%CI 15–49) for those who did not resume treatment (*p* < 0.001).

**Conclusion:**

Delta PS, before and after ICU stay, stands out as a critical determinant of cancer treatment resumption and survival after ICU stay. Multidisciplinary intervention to improve the general condition of these patients, in ICU and after ICU stay, may improve access to cancer treatment and long-term survival.

**Supplementary Information:**

The online version contains supplementary material available at 10.1186/s13613-024-01366-3.

## Background

Although advances in oncology over the past few decades have led to a better prognosis in patients, cancer remains a public health problem and a leading cause of death worldwide [1].

Patients with cancer are exposed to infections, symptoms of cancer progression or drugs’ adverse effects, responsible for intensive care unit (ICU) admission [2, 3]. It is estimated that approximately 5% of patients with cancer may develop critical illness leading to ICU admission within two years of cancer diagnosis, affecting the patient’s outcome [4–6].

Among patients with cancer admitted to the ICU, several factors have been identified as associated with one-year mortality such as metastatic cancer, newly diagnosed cancer at ICU admission, cancer in progression under treatment, poor performance status (PS) and inability to receive oncologic treatment after ICU discharge [3, 7].

Post-intensive care syndrome, implying new or worsened impairments in physical, cognitive, and mental health, could be responsible for inability to receive full cancer treatment after ICU stay in patients with cancer [8]. This may be consequence of altered performance status and persistent organ dysfunction. Thus, a common fear among intensivists and oncologists is that ICU stay, especially when multiple organ support is required, will prevent further treatment of the cancer. However, no study has assessed the risk factors associated with the inability to receive cancer treatment after ICU stay in patients. Accordingly, we conducted a retrospective study to investigate the oncologic outcome of patients with solid tumors after ICU stay. The primary objective was to determine the factors associated with the resumption of cancer treatment after ICU stay in patients with cancer. The secondary objective was to determine the impact of cancer treatment on the long-term outcome of patients with cancer after ICU stay.

## Methods

We conducted a retrospective single-centre study in a 30-bed general medical ICU located in a French University-affiliated Hospital. We included all patients with solid tumors admitted to the ICU between 2014 and 2019. Patients in complete remission from cancer were not included. Patients with treatment-limitation decision at ICU admission were excluded. Regarding patients with several ICU admissions, only the first ICU stay was considered. This work was approved by our institutional ethics committee (number 20.02).

Patient data were obtained retrospectively from electronic medical files. At ICU admission, age, Eastern Cooperative Oncology Group performance status (ECOG PS) [9], medical history, Simplified Acute Physiological Score II (SAPS II) [10], shock and infection were collected. Septic shock was defined according to the Sepsis-3 definition [11]. The cancer history was summarized by the following data: date of diagnosis, diagnosis in ICU, type of cancer, metastatic disease, cancer treatments received (chemotherapy, surgery, radiotherapy, immunotherapy, targeted therapy and/or hormotherapy), number of lines received, ongoing cancer treatment and its type. Cancer treatment was considered ongoing if it had been administered within the 2 months preceding ICU admission. The ICU stay was summarized by the following data: length of stay, reason for admission, maximum number of organ failures [12, 13] (as defined by the SOFA score [14] excluding thrombocytopenia which could be induced by treatment), maximum number of organ replacements, invasive or non-invasive mechanical ventilation, vasopressor support, renal-replacement therapy, extracorporeal membrane oxygenation, acute respiratory distress syndrome, cancer treatment during ICU stay and its type. After the ICU stay, we collected the following data: PS after ICU stay, cancer treatment administration (any systemic or local cancer treatment introduced after the ICU stay) and its type (chemotherapy, surgery, radiotherapy, immunotherapy, targeted therapy and/or hormotherapy), treatment adjustment (protocol chosen due to expected lower toxicity, dose reduction, early discontinuation of the treatment), tumor response to treatment (defined as stable disease, partial response, or complete response at the time of oncology assessment after cancer treatment introduction), date of first progression, outcome, cause of death, cancer status at death. An imaging assessment was systematically performed before resuming cancer treatment. Additionally, imaging assessments of treatment response were conducted every three months from the start of treatment.

Poor PS was defined as ECOG PS of 3 (capable of only limited selfcare; confined to bed or chair more than 50% of waking hours) or 4 (completely disabled; cannot carry on any selfcare; totally confined to bed or chair) [9]. The delta PS was defined as the difference between the PS after ICU stay and the PS before ICU stay. PS before ICU stay was obtained from the most recent report by the referring oncologist, completed within three months before admission. PS after ICU stay was collected within one week of ICU discharge.

### Statistical analyses

Descriptive statistics were used to describe the study population. Patients who received cancer treatment after ICU stay and those who did not receive treatment were compared using Chi-square or Fisher Exact test, as appropriate, for categorical variables, or by Student t-test or Wilcoxon-Mann Whitney test, as appropriate, for continuous variables. A paired samples Wilcoxon test was employed to describe the evolution of PS before and after ICU stay in patients who survived at ICU discharge. Variables associated with cancer treatment resumption after ICU stay in univariable analysis with *p* < 0.1 were then entered into a multivariable logistic regression model after testing for collinearity. The length of invasive mechanical ventilation was excluded, while the length of stay was retained. An alluvial diagram was created to illustrate the resumption of cancer treatment based on the evolution of PS. Survival analysis was performed. Overall survival was defined as the duration from the date of ICU admission to death. Variables associated with survival in univariable analysis with *p* < 0.1 were entered into a Cox proportional hazards model after testing for collinearity and confirming the proportional hazards assumption. The cancer treatment resumption was excluded from the multivariable analysis model to minimize potential confounding biases. Survival rate according to cancer treatment resumption after ICU stay was described by using the Kaplan–Meier method. Variables associated with poor PS after ICU stay in univariable analysis with *p* < 0.1 were then entered into a multivariable logistic regression model after testing for collinearity. The length of invasive mechanical ventilation was excluded from the multivariable analysis model to minimize the effect of collinearity. The first-degree error alpha was fixed to 0.05 bilaterally. Statistical analysis was performed using ‘R’ statistical software.

## Results

### Overall population characteristics

Between 2014 and 2019, 219 patients with solid tumors were admitted to the ICU. Main characteristics of the study population are represented in the Table 1. Of note, 32 (16.2%) patients had a poor ECOG PS at admission (3 or 4). Tumors were mostly non-small cell lung (*n* = 51 [23.6%]), colorectal (*n* = 23 [10.6%]), breast (*n* = 17 [7.9%]), head and neck (*n* = 15 [6.9%]), esophageal (*n* = 13 [6.0%]) and prostate (*n* = 12 [5.6%]) cancers. Cancer treatment was ongoing in 81 (37.2%) of patients upon admission. Among the admitted patients, 68 (32.2%) were diagnosed with cancer during their ICU stay, while 48 (22.0%) had a confirmed cancer diagnosis but had not yet initiated first-line treatment. Furthermore, 21 patients had not undergone treatment in the 2 months preceding ICU admission due to a therapeutic pause. Main causes for ICU admission were acute respiratory failure (*n* = 75 [34.4%]), septic shock (*n* = 40 [18.3%]), cardiac arrest (*n* = 15 [6.9%]), status epilepticus (*n* = 13 [6.0%]), acute kidney injury (*n* = 12 [5.5%]), coma (*n* = 11 [5.0%]) and hemoptysis (*n* = 11 [5.0%]). Forty-eight (44.5%) patients had a poor ECOG PS after ICU stay. The performance status demonstrated statistically significant decline following the ICU stay (median difference − 1.5, 95% confidence interval CI [-1.5-1.0], *p* < 0.001).

**Table 1:** Characteristics of overall population

Characteristics	Overall population (*n* = 219)
**Male sex**,** n (%)**	149 (68.0)
**Age at ICU admission**,** median [IQR]**	63 [54, 69]
**Poor performance status (3–4) before ICU stay**,** n (%)** ***Missing data = 22***	32 (16.2)
**Details of performance status**,** n (%)**	
0	35 (17.8)
1	100 (50.8)
2	30 (15.2)
3	30 (15.2)
4	2 (1.0)
**Sites of cancer**,** n (%)** ***Missing data = 3***	
Non-small cell lung cancer	51 (23.6)
Colorectal	23 (10.6)
Breast	17 (7.9)
Head and neck	15 (6.9)
Esophageal	13 (6.0)
Prostate	12 (5.6)
Carcinoma of unknown primary	10 (4.6)
Small cell lung cancer	8 (3.7)
Kidney	7 (3.2)
Bladder	7 (3.2)
Ovarian	7 (3.2)
Glioblastoma	7 (3.2)
Testis	6 (2.8)
Melanoma	5 (2.3)
Others	28 (13.1)
**Time from cancer diagnosis to ICU admission (months)**,** median [IQR]**	4 [1, 20]
**Metastatic disease**,** n (%)** ***Missing data = 5***	141 (65.9)
**Treatment received before ICU**,** n (%)**	
Radiotherapy	63 (28.8)
Chemotherapy	91 (41.6)
Immune checkpoint inhibitors	7 (3.2)
Targeted therapy	19 (8.7)
Hormonotherapy	18 (8.2)
**Number of treatment lines before ICU**,** median [IQR]** ***Missing data = 1***	1 [0, 1]
***Details***	
0	116 (53.2)
1	70 (32.1)
2	16 (7.3)
3	11 (5.0)
4	2 (0.9)
5	1 (0.5)
7	2 (0.9)
**Ongoing cancer treatment**,** n (%)** ***Missing data = 1***	81 (37.2)
***Type of treatment***,*** n (%)*** ***Missing data = 2***	
Chemotherapy	42 (53.8)
Hormonotherapy	5 (6.4)
Targeted therapy	7 (9.0)
Immune checkpoint inhibitors	3 (3.8)
Radiotherapy and chemotherapy	10 (12.8)
Chemotherapy and targeted therapy	10 (12.8)
Chemotherapy and hormonotherapy	1 (1.3)
**SAPS II**,** median [IQR]** ***Missing data = 3***	44.0 [32.8, 66.3]
**Cause for ICU admission**,** n (%)** ***Missing data = 1***	
Acute respiratory failure	75 (34.4)
Septic shock	40 (18.3)
Cardiac arrest	15 (6.9)
Status epilepticus	13 (6.0)
Acute kidney injury	12 (5.5)
Coma	11 (5.0)
Hemoptysis	11 (5.0)
Sepsis	9 (4.1)
Cardiogenic shock	6 (2.8)
Others	26 (12.4)
**Diagnosis of cancer in ICU**,** n (%)**	68 (31.1)
**Cancer treatment during ICU stay**,** n (%)** ***Missing data = 1***	13 (6.0)
***Type of treatment***,*** n (%)***	
Chemotherapy	6 (46.2)
Targeted therapy	2 (15.4)
Surgery	5 (38.5)
**Shock**,** n (%)**	84 (38.4)
**Infection at ICU admission**,** n (%)**	116 (53.0)
**Site of infection**,** n (%)** ***Missing data = 3***	
Respiratory	63 (56.3)
Cutaneous	1 (0.9)
Urinary	12 (10.7)
Digestive	14 (12.5)
Bloodstream infection	5 (4.5)
Catheter-related bloodstream infection	6 (5.4)
Fungemia	1 (0.9)
Others	11 (9.9)
**Maximum number of organ failures**,** median [IQR]** ***Missing data = 2***	2 [1, 3]
**Maximum number of organ replacements**,** median [IQR]** ***Missing data = 3***	1 [0, 2]
**Invasive mechanical ventilation**,** n (%)** ***Missing data = 1***	131 (60.1)
**Non invasive ventilation**,** n (%)** ***Missing data = 1***	17 (7.8)
**High-flow nasal cannula therapy**,** n (%)** ***Missing data = 1***	9 (4.1)
**Vasopressor support**,** n (%)** ***Missing data = 1***	99 (45.4)
**Renal-replacement therapy**,** n (%)** ***Missing data = 3***	30 (13.9)
**V-A ECMO**,** n (%)** ***Missing data = 1***	1 (0.5)
**V-V ECMO**,** n (%)** ***Missing data = 1***	2 (0.9)
**Acute respiratory distress syndrome**,** n (%)** ***Missing data = 1***	15 (6.9)
**Length of invasive mechanical ventilation (days)**, **median [IQR]** ***Missing data = 1***	5.5 [2.0, 10.0]
**Length of stay (days)**,** median [IQR]**	5 [2, 9]
**Poor performance status (3–4) after ICU stay**,** n (%)** ***Missing data = 28***	48 (44.5)
**Details of performance status**,** n (%)**	
0	3 (2.8)
1	40 (37.0)
2	17 (15.7)
3	29 (26.9)
4	19 (17.6)

IQR: interquartile range. ICU: intensive care unit. SAPS II: Simplified Acute Physiological Score II. V-A ECMO: veno-arterial extracorporeal membrane oxygenation. V-V ECMO: veno-venous extracorporeal membrane oxygenation

### Factors associated with cancer treatment resumption after ICU stay

Among the 136 patients who survived the ICU stay, 81 (59.6%) received cancer treatment after ICU discharge. The main treatments were chemotherapy (*n* = 32 [39.5%]), surgery (*n* = 11 [13.6%]), radiotherapy (*n* = 9 [11.1%]), hormonotherapy (*n* = 7 [8.6%]) or immune checkpoint inhibitors (*n* = 5 [6.2%]). A treatment adjustment was made for 19 (30.2%) patients. Tumor response to treatment (defined as stable disease, partial response, or complete response at the time of oncology assessment after cancer treatment introduction) was observed in 57 (70.4%) patients, with the best overall response being complete response in 19 (33.3%) patients, partial response in 25 (43.9%), and stable disease in 10 (17.5%). Following the initiation of treatment after the ICU stay, 16 patients (21.9%) experienced disease progression without any tumor response.

Characteristics of the population according to cancer treatment resumption and univariable analysis are shown in the Table 2. A logistic regression model was used to explore the association of infection, length of stay, maximum number of organ failures, maximum number of organ replacements and delta PS, with cancer treatment resumption after ICU stay. At multivariable analysis, delta PS (Odds ratio OR 0.34, 95%CI 0.18–0.56, p value < 0.001) was independently associated with inability to receive cancer treatment (Table 3).

**Table 2:** Characteristics of population according to cancer treatment resumption and univariable analysis

Characteristics	No treatment after ICU (*n* = 49)	Treatment after ICU (*n* = 81)	*p*
**Male sex**,** n (%)**	33 (67.3)	59 (72.8)	0.553
**Age at ICU admission**,** median [IQR]**	64 [56, 72]	63 [53, 68]	0.231
**Poor performance status (3–4) before ICU stay**,** n (%)** ***Missing data = 22***	9 (20)	5 (6.6)	**0.040**
**Details of performance status**,** n (%)**			
0	5 (11.1)	21 (27.6)	
1	23 (51.1)	39 (51.3)	
2	8 (17.8)	11 (14.5)	
3	8 (17.8)	5 (6.6)	
4	1 (2.2)	0 (0.0)	
**Sites of cancer**,** n (%)** ***Missing data = 3***			0.181
Non-small cell lung cancer	11 (22.9)	18 (22.2)	
Colorectal	7 (14.6)	7 (8.6)	
Breast	3 (6.2)	7 (8.6)	
Head and neck	0 (0.0)	9 (11.1)	
Esophageal	7 (14.6)	2 (2.5)	
Prostate	2 (4.2)	7 (8.6)	
Carcinoma of unknown primary	3 (6.2)	1 (1.2)	
Small cell lung cancer	1 (2.1)	2 (2.5)	
Kidney	1 (2.1)	2 (2.5)	
Bladder	2 (4.2)	2 (2.5)	
Ovarian	1 (2.1)	2 (2.5)	
Glioblastoma	1 (2.1)	5 (6.2)	
Testis	1 (2.1)	2 (2.5)	
Melanoma	1 (2.1)	2 (2.5)	
Others	7 (14.7)	13 (16.0)	
**Metastatic disease**,** n (%)** ***Missing data = 5***	30 (61.2)	45 (56.2)	0.713
**Treatment received before ICU**,** n (%)**			
Radiotherapy	15 (30.6)	24 (29.6)	1.000
Chemotherapy	23 (46.9)	30 (37.0)	0.276
Immune checkpoint inhibitors	1 (2.0)	4 (4.9)	0.649
Targeted therapy	7 (14.3)	7 (8.6)	0.385
Hormonotherapy	3 (6.1)	7 (8.6)	0.742
**Number of treatment lines before ICU**,** median [IQR]** ***Missing data = 1***	0 [0, 1]	1 [0, 1]	0.683
**Ongoing cancer treatment**,** n (%)** ***Missing data = 1***	15 (31.2)	29 (35.8)	0.702
***Type of treatment***,*** n (%)*** ***Missing data = 1***			0.210
Chemotherapy	5 (33.3)	13 (48.1)	
Hormonotherapy	0 (0.0)	2 (7.4)	
Targeted therapy	2 (13.3)	0 (0.0)	
Immune checkpoint inhibitors	0 (0.0)	3 (11.1)	
Radiotherapy and chemotherapy	4 (26.7)	4 (14.8)	
Chemotherapy and targeted therapy	4 (26.7)	4 (14.8)	
Chemotherapy and hormonotherapy	0 (0.0)	1 (3.7)	
**SAPS II**,** median [IQR]** ***Missing data = 3***	41.0 [31.0, 56.0]	37.0 [28.8, 47.0]	0.129
**Cause for ICU admission**,** n (%)** ***Missing data = 1***			0.330
Acute respiratory failure	20 (40.8)	21 (26.2)	
Septic shock	11 (22.4)	8 (10.0)	
Cardiac arrest	3 (6.1)	2 (2.5)	
Status epilepticus	3 (6.1)	9 (11.2)	
Acute kidney injury	2 (4.1)	7 (8.8)	
Coma	0 (0.0)	7 (8.8)	
Hemoptysis	2 (4.1)	5 (6.2)	
Infection without shock	3 (6.1)	6 (7.5)	
Cardiogenic shock	0 (0.0)	1 (1.2)	
Others	5 (10.1)	14 (17.3)	
**Diagnosis of cancer in ICU**,** n (%)**	15 (30.6)	21 (25.9)	0.686
**Cancer treatment during ICU stay**,** n (%)** ***Missing data = 1***	2 (4.1)	4 (5.0)	1.000
***Type of treatment***,*** n (%)***			1.000
Chemotherapy	1 (50.0)	1 (25.0)	
Targeted therapy	0 (0.0)	0 (0.0)	
Surgery	1 (50.0)	3 (75.0)	
**Shock**,** n (%)**	14 (28.6)	21 (25.9)	0.839
**Infection**,** n (%)**	31 (63.3)	37 (45.7)	**0.070**
**Site of infection**,** n (%)** ***Missing data = 3***			0.331
Respiratory	17 (54.8)	17 (47.3)	
Cutaneous	1 (3.2)	0 (0.0)	
Urinary	5 (16.2)	7 (19.4)	
Digestive	2 (6.5)	3 (8.3)	
Bloodstream infection	1 (3.2)	1 (2.8)	
Catheter-related bloodstream infection	0 (0.0)	3 (8.4)	
Fungemia	1 (3.2)	0 (0.0)	
Others	4 (12.8)	5 (14)	
**Maximum number of organ failures**,** median [IQR]** ***Missing data = 2***	2 [1, 3]	1 [1, 2]	**0.005**
**Maximum number of organ replacements**,** median [IQR]** ***Missing data = 3***	1 [1, 1]	1 [0, 1]	**0.017**
**Invasive mechanical ventilation**,** n (%)** ***Missing data = 1***	24 (49.0)	32 (40.0)	0.362
**Non invasive ventilation**,** n (%)** ***Missing data = 1***	7 (14.3)	4 (5.0)	0.102
**High-flow nasal cannula therapy**,** n (%)** ***Missing data = 1***	5 (10.2)	2 (2.5)	0.104
**Vasopressor support**,** n (%)** ***Missing data = 1***	18 (36.7)	19 (23.8)	0.160
**Renal-replacement therapy**,** n (%)** ***Missing data = 3***	6 (12.2)	4 (5.0)	0.178
**V-A ECMO**,** n (%)** ***Missing data = 1***	0 (0.0)	0 (0.0)	1.000
**V-V ECMO**,** n (%)** ***Missing data = 1***	0 (0.0)	1 (1.2)	1.000
**Acute respiratory distress syndrome**,** n (%)** ***Missing data = 1***	2 (4.1)	1 (1.2)	0.557
**Length of invasive mechanical ventilation (days)**, **median [IQR]** ***Missing data = 1***	9.0 [4.0, 11.0]	3.0 [1.0, 7.0]	**0.004**
**Length of stay (days)**,** median [IQR]**	6 [2, 11]	4 [2, 6]	**0.041**
**Poor performance status (3–4) after ICU stay**,** n (%)** ***Missing data = 28***	32 (82.1)	15 (22.4)	**< 0.001**
**Details of performance status**,** n (%)**			
0	1 (2.6)	2 (3.0)	
1	2 (5.1)	37 (55.2)	
2	4 (10.3)	13 (19.4)	
3	15 (38.5)	14 (20.9)	
4	17 (43.6)	1 (1.5)	

IQR: interquartile range. ICU: intensive care unit. SAPS II: Simplified Acute Physiological Score II. V-A ECMO: veno-arterial extracorporeal membrane oxygenation. V-V ECMO: veno-venous extracorporeal membrane oxygenation

**Table 3:** Multivariable analysis of factors associated with treatment resumption

Variables	Multivariable analysis	
	OR (95% CI)	*p*
Infection	0.79 (0.26–2.37)	0.666
Length of stay	0.99 (0.92–1.07)	0.863
Maximum number of organ failures	0.90 (0.51–1.61)	0.728
Maximum number of organ replacements	1.12 (0.44–2.91)	0.807
**Delta PS**	**0.34 (0.18–0.56)**	**< 0.001**

PS: performance status

**Fig. 1:**
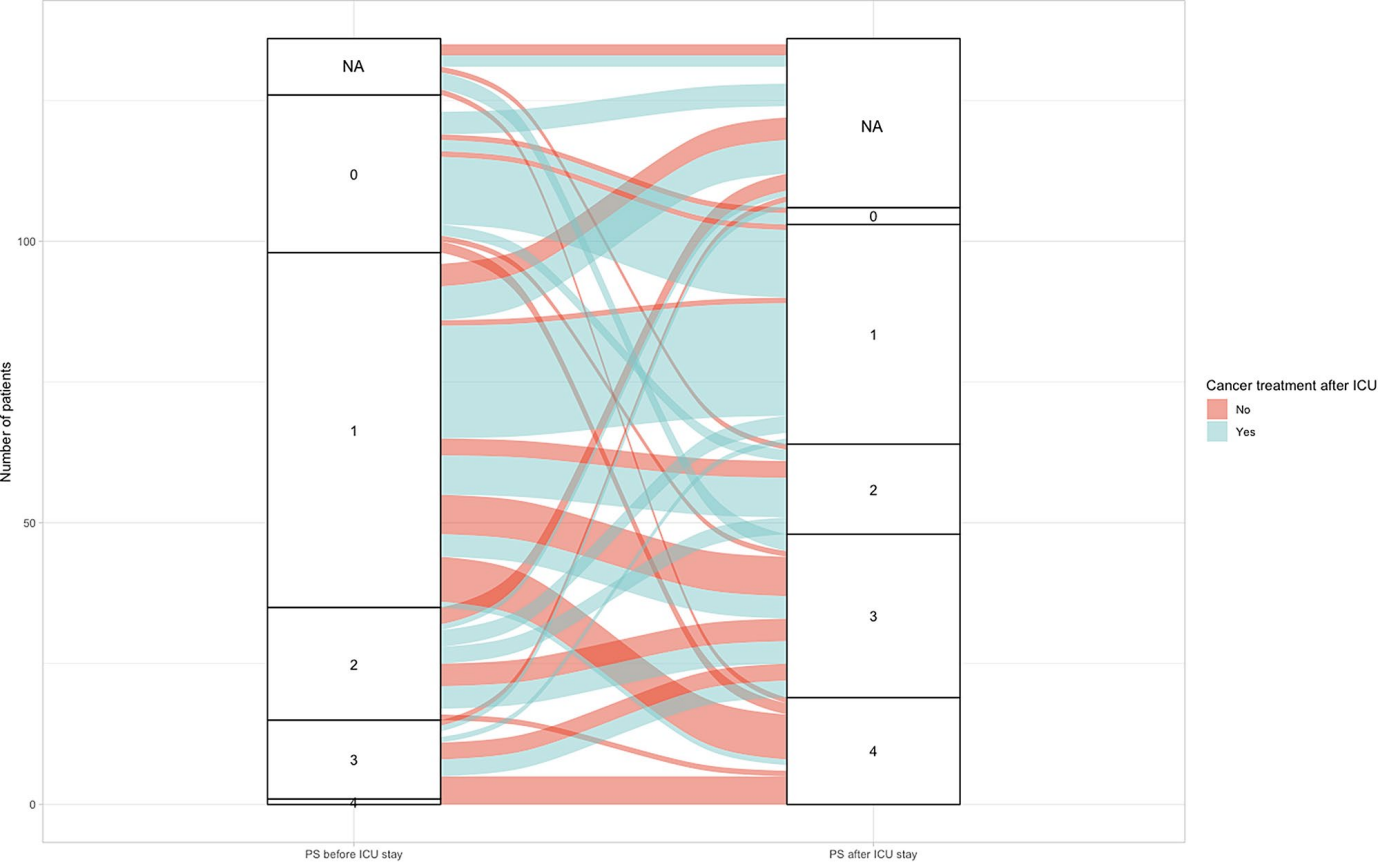
Alluvial diagram illustrating the resumption of cancer treatment based on the evolution of performance status

Figure 1 was designed to illustrate the resumption of cancer treatment based on the evolution of PS.

### Outcome

Survival rates at ICU discharge, at 6 months, at 1, 2 and 3 years were 62.3% (*n* = 136), 35.1% (*n* = 76), 27.3% (*n* = 59), 21.8% (*n* = 47) and 17.1% (*n* = 37), respectively.

The main causes of death in ICU were infection (*n* = 32 [39.0%]), cancer-related (*n* = 28 [34.1%]), cardiac arrest (*n* = 9 [11.0%]), specific toxicity of the cancer treatment (*n* = 5 [6.1%]) and stroke (*n* = 3 [3.7%]). Four patients had treatment limitations during their ICU stay, which was followed by death in the ICU.

After a median follow up of 65 months [interquartile range 49–78] after ICU discharge, 102 (76.7%) patients died. Median overall survival of patients who survived the ICU stay was 9.0 months (95% confidence interval [5.0-12.6]). The majority of deaths after ICU stay were ultimately cancer-related (*n* = 76 [86.4%]), four patients (4.5%) died from infection. At the time of death, cancer remained predominantly active (*n* = 108 [85.7%]), with few patients in remission or cured (*n* = 18 [14.3%]).

A Cox regression model was used to explore the association of delta PS, metastatic disease and diagnosis of cancer in ICU with 1-year mortality in patients who survived at ICU discharge. At multivariable analysis, delta PS (HR 1.76, 95%CI 1.34–2.31, p value < 0.001) was independently associated with 1-year mortality in patients who survived at ICU discharge (Table 4).

**Table 4:** Multivariable analysis of factors associated with 1-year mortality in patients who survived at ICU discharge

Variables	Multivariable analysis	
	HR (95% CI)	*p*
Delta PS	1.76 (1.34–2.31)	< 0.001
Metastatic disease	1.72 (0.95–3.11)	0.072
Diagnosis of cancer in ICU	1.50 (0.82–2.73)	0.185

PS: performance status. ICU: intensive care unit

Another Cox regression model was used to explore the association of delta PS, cardiovascular disease, cirrhosis, shock, diagnosis of cancer in ICU and maximum number of organ replacements, with 3-year mortality in patients who survived at ICU discharge. At multivariable analysis, delta PS (HR 1.86, 95%CI 1.44–2.39, p value < 0.001), cardiovascular disease (HR 0.34, 95%CI 0.17–0.68, p value 0.002) and cirrhosis (HR 2.91, 95%CI 1.13–7.49, p value 0.027) was independently associated with 3-year mortality in patients who survived at ICU discharge.

**Fig. 2:**
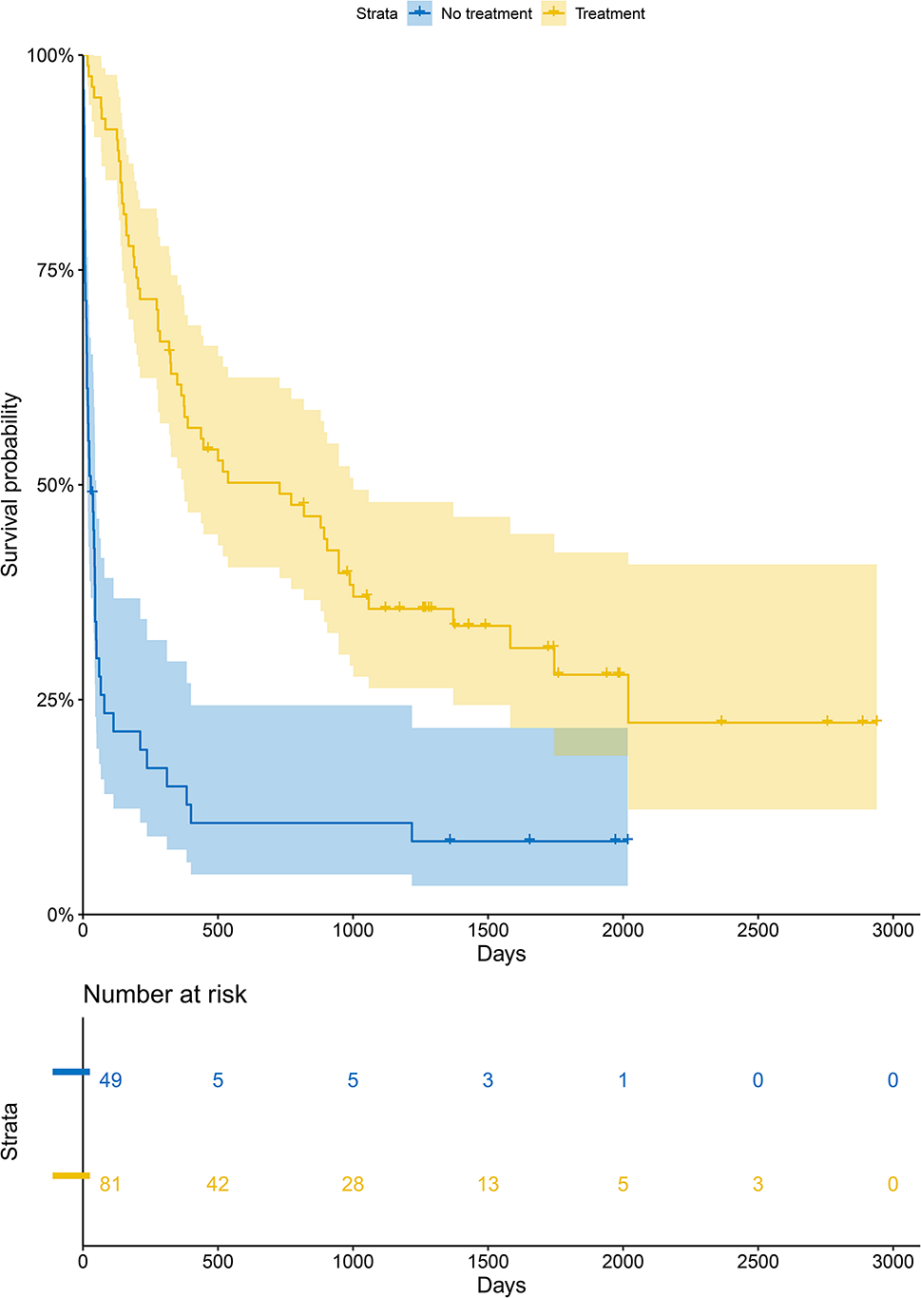
Overall survival curves according to cancer treatment resumption

The survival rate according to cancer treatment resumption after ICU stay was described by using the Kaplan–Meier method (Fig. 2). The median survival for patients who resumed cancer treatment after ICU stay was 771 days (95%CI 376–1058), compared to 29 days (95%CI 15–49) for those who did not resume treatment (*p* < 0.001). Cancer treatment adjustment was not associated with 1-year mortality (*p* = 0.293) or 3-year mortality (*p* = 0.413) in univariate analysis. The patient’s course from ICU admission to treatment resumption was illustrated in a flow chart (supplementary figure S1).

### Factors associated with poor PS after ICU

A logistic regression model was used to explore the association of PS before ICU stay, infection, length of stay, maximum number of organ failures and maximum number of organ replacements, with poor PS after ICU stay. At multivariable analysis, PS before ICU stay (OR 3.73, 95%IC 2.01–7.82, p value < 0.001) and length of stay (OR 1.23, 95%CI 1.06–1.49, p value 0.018) was independently associated with poor PS after ICU stay (Table 5).

**Table 5:** Multivariable analysis of factors associated with poor PS after ICU

Variables	Multivariable analysis	
	OR (95% CI)	*p*
**PS before ICU stay**	**3.73 (2.01–7.82)**	**< 0.001**
Infection	2.29 (0.78–6.96)	0.134
**Length of stay**	**1.23 (1.06–1.49)**	**0.018**
Maximum number of organ failures	1.38 (0.73–2.70)	0.327
Maximum number of organ replacements	0.70 (0.25–1.91)	0.487

PS: performance status. ICU: intensive care unit

## Discussion

In this retrospective study, we found that the change in PS before and after ICU stay (delta PS) was associated with inability to receive cancer treatment. To our knowledge, this is the first study assessing factors associated with cancer treatment resumption after ICU stay. In our study, 59.6% of the patients surviving the ICU stay were receiving cancer treatment after ICU stay. The median survival for patients who resumed cancer treatment after ICU stay was 771 days (95%CI 376–1058), compared to 29 days (95%CI 15–49) for those who did not resume treatment (*p* < 0.001). ICU stay has a tremendous impact on PS as we observed an important increase in patients with poor PS of 3 or 4 after ICU stay (16.2% at ICU admission vs. 44.5% of patients who survived), with statistically significant PS decline following the ICU stay. PS before ICU stay and length of stay was associated with poor PS after ICU stay. Importantly, the main cause of death after ICU stay was cancer related. Moreover, we found that delta PS was associated with 1-year mortality in patients who survived ICU discharge. Additionally, delta PS, along with cardiovascular disease and cirrhosis, was independently associated with 3-year mortality in these patients.

Delta PS was associated with inability to receive cancer treatment after ICU stay, and factors associated with poor PS after ICU stay included PS before ICU stay and the length of ICU stay. On one hand, these elements may inform ethical considerations. They suggest that a cancer patient with impaired PS before ICU admission may experience further deterioration during the ICU stay, potentially hindering the resumption of cancer treatment and affecting survival outcomes. These findings underscore the importance of carefully evaluating the potential benefits and risks of ICU admission for such patients. The impact of PS before ICU stay on cancer patients outcome has been widely demonstrated [15, 16]. However, more than PS at any given time, delta PS may have a greater impact on the resumption of cancer treatment and patient outcome. This change in PS, observable over the course of the ICU stay, could provide valuable insights during ICU trials [17]. On the other hand, preventive strategies to prevent PS decline during the ICU stay, or to improve it afterwards, might impact patient’s outcome. There was an important increase in patients with poor PS of 3 or 4 after ICU stay. PS is a major factor in oncology decision-making, as evidenced by the inclusion criteria for clinical trials that require good general condition [18]. As a result, 59.6% of patients received cancer treatment after ICU stay. García de Herreros et al. also demonstrated a significant impact of ICU stay on PS, with 40% of survivors experiencing permanent discontinuation of cancer treatment [19]. While active mobilization and rehabilitation in the ICU have shown potential benefits for improving mobility and muscle strength in general population, the results of this type of intervention remain mixed and require further exploration [20, 21]. Notably, active physiotherapy in the ICU for intubated patients with malignancy has been demonstrated to be feasible and safe [22]. However, an individualized eight-week home-based physical rehabilitation program did not increase the underlying rate of recovery after ICU stay, with both groups of critically ill survivors improving their physical function over the 26 weeks of follow-up [23]. To date, no trial has assessed the effectiveness of combined nutritional and physical rehabilitation initiated in the ICU and continued after ICU stay, either in the general population or specifically in cancer patients. Interestingly, Gheerbrant et al. showed the evolution of PS over time in survivors with 20.2% of patients with poor PS at admission versus 12.7% at 3 months and 8.2% at 6 months. At 3 months, 55% of patients received cancer treatment [15].

The median survival for patients who resumed cancer treatment after ICU stay was 771 days, compared to 29 days for those who did not resume treatment. The observed difference in survival seems likely to be due to the early mortality of patients who do not resume treatment, rather than the effect of resuming cancer treatment itself, as 50% of these patients die within the month following ICU discharge. In our study, cancer patients who resumed treatment after ICU stay had prolonged survival. Resuming cancer treatment in these patients may significantly improve survival by controlling the underlying disease. Their prognosis after ICU stay appears to be mainly related to the cancer evolution. Conversely, those who did not resume treatment had a median survival of less than one month, with approximately 80% in a compromised general condition, making the resumption of treatment unlikely in this population. Their prognosis appears to be more related to the acute event leading to ICU admission rather than the cancer itself. The study suggests that the long-term mortality of patients may also be linked to their comorbidities, potentially stemming from either the impediment to receiving optimal cancer treatment or complications directly arising from the comorbidity itself [24]. Noteworthy, patient survival in our study was lower compared to the literature [3, 7]. This could be attributed to the inclusion criteria that specifically targeted patients with cancer in place, who may present more severe conditions. At ICU discharge, the prognosis of these patients might be worse because of the cancer in place. Gheerbrant et al. showed that 29% of patients had no indication for cancer treatment at 3 months after ICU discharge in a study allowing the inclusion of patients with cancer in remission for less than 5 years [15]. This suggests that many patients might be cured.

Therefore, ICU stay alters general condition and probably limit but does not prevent cancer treatment resumption. This should not prevent the patient from being admitted in ICU. The evolution of PS from ICU admission to discharge stands out as a critical determinant of oncologic outcomes, especially regarding cancer treatment resumption and long-term survival. The patient’s overall condition, especially its trajectory throughout their ICU stay, could significantly inform ethical considerations regarding the care of these individuals. Implementing comprehensive specialized management, encompassing aspects such as nutrition, physical rehabilitation, psychological support, emerges as a crucial component for facilitating the resumption of cancer treatments and enhancing the survival of these patients after ICU stay [25, 26].

This study has several strengths. Noteworthy, the general characteristics of our study population were in line with the literature [3]. The predominant cancer types in our study population were consistent with cancers epidemiology in Europe, except for head and neck and esophageal cancers which are overrepresented [27]. The higher level of comorbidities in some patients with head and neck and esophageal cancers, or the more frequent occurrence of respiratory complications in these patients, may provide an explanation for these results [28–30]. Notably, this study is the first to assess factors associated with the resumption of cancer treatment after an ICU stay in patients with cancer. It adopts a pragmatic approach, aiming to assist physicians in decision-making when confronted with complex medical and ethical situations. Cured patients or patients in remission were not included in the study, which allowed to meet the main objective, to focus on the more complex situations and to avoid overestimating the survival of patients with cancer admitted to the ICU. Patients with treatment-limitation decisions at ICU admission were excluded from the study. Significant variability in ICU triage decisions for cancer patients has been documented [31]. Admission policies differ across centers, with some admitting few or no cancer patients with treatment limitations due to their prognosis impact. Excluding these patients aids in meeting the primary objective by avoiding confounding factors, as this specific group often has more compromised conditions and oncological treatment restrictions. This exclusion also enhances the generalizability of our results. However, the study also has several limitations. Firstly, it is a single-center study, which may limit the generalizability of our results. Secondly, while our patient selection criteria are designed to meet our objectives by minimizing known confounding factors, they consequently select for a population with high proportion of patients diagnosed either in ICU or recently diagnosed, and do not provide information on patients with treatment-limitation decision. Thirdly, the retrospective nature of the study introduces potential biases. Certain data are missing, such as disease status (controlled disease, relapse, or progression) at the time of ICU admission or whether ICU admission was due to specific cancer treatment toxicity, potentially introducing confounding bias. Lastly, patients were included from 2014 to 2019. Oncology is undergoing a major therapeutic revolution across time, which means that cancer treatments change rapidly over time but also that patient prognosis may change accordingly.

## Conclusion

Delta PS, before and after ICU stay, was independently associated with inability to receive cancer treatment, and with long-term mortality in patients who survived at ICU discharge. There was an important increase in patients with poor PS of 3 or 4 after ICU stay. More than half of the patients surviving the ICU stay were receiving cancer treatment after ICU stay. The median survival for patients who resumed cancer treatment after ICU stay was 771 days, compared to 29 days for those who did not resume treatment. Outcome of patients with cancer after ICU stay may be determined by their general condition and their oncological outcome. These findings can provide valuable insights for ethical considerations both before ICU admission and throughout the patient’s stay. Special attention should be paid to these patients at ICU discharge for comprehensive evaluation. Multidisciplinary intervention to improve the general condition of these patients may improve access to cancer treatment and long-term survival.

### Electronic Supplementary Material

Below is the link to the electronic supplementary material.

Supplementary Material 1

## Data Availability

The datasets used and/or analyzed during the current study are available from the corresponding author on reasonable request.

## Abbreviations

ICU: Intensive care unit
ST: Solid tumors
PS: Performance status
OR: Odds ratio
HR: Hazard ratio
ECOG PS: Eastern Cooperative Oncology Group performance status
SAPS II: Simplified Acute Physiological Score II
