# Supplementary material for: Factors associated with cancer treatment resumption after ICU stay in patients with solid tumors
Source: Ann Intensive Care. 2024 Aug 31;14:135. doi: 10.1186/s13613-024-01366-3 (PMC11365869; doi:10.1186/s13613-024-01366-3)
Supplement: Supplementary file 1 — Supplementary Material 1 [file 13613_2024_1366_MOESM1_ESM.pptx]

## Slide 1
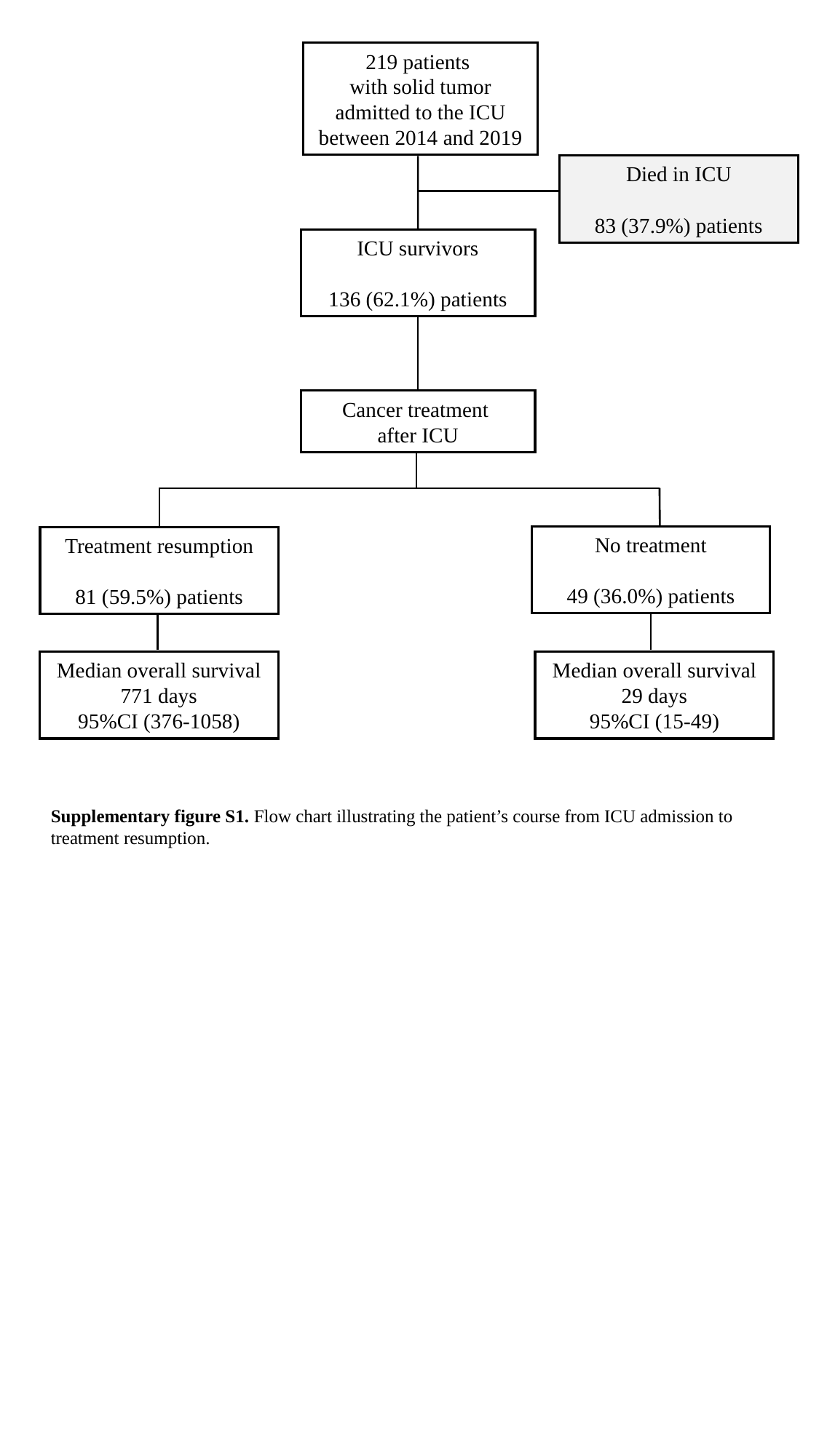

219 patients
with solid tumor admitted to the ICU between 2014 and 2019
Died in ICU
83 (37.9%) patients
ICU survivors
136 (62.1%) patients
Cancer treatment
after ICU
No treatment
49 (36.0%) patients
Treatment resumption
81 (59.5%) patients
Median overall survival
771 days
95%CI (376-1058)
Median overall survival
29 days
95%CI (15-49)
Supplementary figure S1. Flow chart illustrating the patient’s course from ICU admission to treatment resumption.
